# Population prevalence of individuals meeting criteria for hereditary breast and ovarian cancer testing

**DOI:** 10.1002/cam4.2534

**Published:** 2019-09-18

**Authors:** Samantha Greenberg, Saundra S. Buys, Sandra L. Edwards, Whitney Espinel, Alison Fraser, Amanda Gammon, Brent Hafen, Kimberly A. Herget, Wendy Kohlmann, Camille Roundy, Carol Sweeney

**Affiliations:** ^1^ Huntsman Cancer Institute University of Utah Salt Lake City Utah; ^2^ Department of Internal Medicine University of Utah Salt Lake City Utah; ^3^ Utah Cancer Registry University of Utah Salt Lake City Utah; ^4^ Intermountain Healthcare Salt Lake City Utah; ^5^ Utah Department of Health Salt Lake City Utah

**Keywords:** BRCA1/2, cancer registry, epidemiology, genetic counseling, hereditary cancer

## Abstract

**Background:**

Personal cancer diagnosis and family cancer history factor into which individuals should undergo genetic testing for hereditary breast and ovarian cancer (HBOC) syndrome. Family history is often determined in the research setting through kindreds with disease clusters, or clinically from self‐report. The population prevalence of individuals with diagnostic characteristics and/or family cancer history meeting criteria for HBOC testing is unknown.

**Methods:**

Utilizing Surveillance, Epidemiology, and End Results (SEER) cancer registry data and a research resource linking registry records to genealogies, the Utah Population Database, the population‐based prevalence of diagnostic and family history characteristics meeting National Comprehensive Cancer Network (NCCN) criteria for HBOC testing was objectively assessed.

**Results:**

Among Utah residents with an incident breast cancer diagnosis 2010‐2015 and evaluable for family history, 21.6% met criteria for testing based on diagnostic characteristics, but the proportion increased to 62.9% when family history was evaluated. The proportion of cases meeting testing criteria at diagnosis was 94% for ovarian cancer, 23% for prostate cancer, and 51.1% for pancreatic cancer. Among an unaffected Utah population of approximately 1.7 million evaluable for family history, 197,601 or 11.6% met testing criteria based on family history.

**Conclusions:**

This study quantifies the population‐based prevalence of HBOC criteria using objectively determined genealogy and cancer incidence data. Sporadic breast cancer likely represents a portion of the high prevalence of family cancer history seen in this study. These results underline the importance of establishing presence of a deleterious mutation in an affected family member, per NCCN guidelines, before testing unaffected relatives.

## Graphical abstract

Using diagnostic and family history data, almost 63% of individuals with breast cancer diagnoses meet criteria for genetic testing based on diagnostic and family history. Utilizing the Utah Population Database, a research resource that links of four decades of cancer registry records to genealogies, almost 12% of the unaffected Utah population meets criteria for genetic testing. Given the high proportion of the population, targeting affected cases can maximize family impact while minimizing cost.

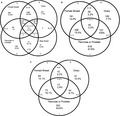

## 1 INTRODUCTION

Hereditary breast and ovarian cancer (HBOC) caused by pathogenic variants in *BRCA1* or *BRCA2* represent approximately 2% of breast cancers^1^ and 10%‐15% of ovarian cancers.^2^, ^3^ Clinical counseling and testing services for hereditary breast and ovarian cancer have been available since the 1990s and criteria for identifying breast and ovarian cancer cases due to hereditary causes have been developed over time. Several characteristics associated with an increased likelihood of a pathogenic *BRCA1* or *BRCA2* variant include young age of breast cancer onset (<45 years), epithelial ovarian cancer, triple negative breast cancer, or multiple relatives with breast and/or ovarian cancer. Women with a *BRCA1 or BRCA2* pathogenic variant have a 50%‐80% risk of developing breast cancer and a 20%‐40% risk of developing ovarian cancer. Men with *BRCA1* or *BRCA2* pathogenic variants have a 15%‐30% risk of prostate cancer and a 2%‐6% risk of male breast cancer.^4^ When HBOC pathogenic variant status is known, the risk of cancer mortality can be reduced by following established management guidelines including cancer screening at younger ages or risk‐reducing mastectomy and salpingo‐oophorectomy.

Given the opportunity for reduced morbidity and mortality, The Centers for Disease Control and Prevention (CDC)'s Office of Public Health Genomics has identified *BRCA1/*2 testing as a genomic application that has potential for positive impact on the public's health.^5^, ^6^ Guidelines on who should receive genetic counseling and testing have been compiled by expert committees,^7^, ^8^, ^9^ and are repeatedly revised and expanded. Recent guidelines have suggested more widespread genetic testing given that people with pathogenic variants may not meet referral criteria.^10^, ^11^ A widely used set of criteria are disseminated by the National Comprehensive Cancer Network (NCCN). However, there is limited understanding of the prevalence of individuals who meet various testing criteria and thus of the influence of criteria on number of people who might be recommended for testing. Cancer registry data can be used to evaluate patients who meet NCCN criteria based on the diagnostic characteristics of their cancer such as age at diagnosis, epithelial ovarian cancer histology, and triple‐negative breast cancer. HER2 status, a variable needed to define triple‐negative breast cancer, has been available in cancer registry data only since 2010. To the best of our knowledge, the population‐based prevalence of cancers meeting testing criteria due to diagnostic characteristics has not recently been summarized.

Family cancer history information is a key component of testing criteria both for individuals affected with cancer and for unaffected patients. Cancer registry databases do not include information about family history of cancer.^6^ Family history is often determined in the research setting through kindreds with recognized clusters of HBOC diagnoses. In the clinical setting, family history is obtained from self‐report. The validity of self‐report of family history is a concern and may vary based on subject characteristics.^12^ Population‐based data on family history of cancer has been compiled from self‐report on national surveys; however, the detail needed to apply HBOC testing criteria is not available.^13^ Collection of high‐quality self‐reported family history data requires specialized tools and is not widely implemented.^14^ The Utah Population Database (UPDB) is a research resource that links over four decades of state‐wide central cancer registry records to extensive genealogies, allowing for objective assessment of family history of cancer for both affected and unaffected individuals. This study seeks to estimate the population prevalence of individuals meeting criteria for HBOC and genetic testing based on the characteristics of a personal diagnosis of cancer, and based on the combination of personal and family cancer history.

## 2 MATERIALS AND METHODS

### 2.1 Research design and study population

We used data from the US National Cancer Institute's Surveillance, Epidemiology, and End Results (SEER) program SEER 18 Registries^15^ to calculate the prevalence of diagnostic characteristics meeting NCCN HBOC testing criteria among a set of incident cancers representing a broad sample of the US diagnosed during the period 2010‐2015. Testing criteria defined in the National Comprehensive Cancer Network (NCCN) Familial Breast/Ovarian Risk Assessment guidelines was used (V2.2015, Figure 1). Cancer‐specific variables including cancer site, histology, SEER summary stage, year of diagnosis, diagnostic information, ethnicity, and age at diagnosis were queried from SEER. NCCN criteria evaluable from SEER data include male breast cancer, young age at diagnosis, triple‐negative breast cancer (estrogen receptor, progesterone receptor, and human epidermal growth factor receptor 2 amplification negative), second primary diagnosis of breast cancer, and diagnosis of ovarian, primary peritoneal, or fallopian tube cancer. Breast cancers considered included all invasive diagnoses and ductal carcinoma in situ (DCIS). Other cancer sites were limited to invasive diagnoses. Specific cancer site and histology codes that were used to establish diagnoses meeting criteria can be found in Table S1.

**Figure 1:**
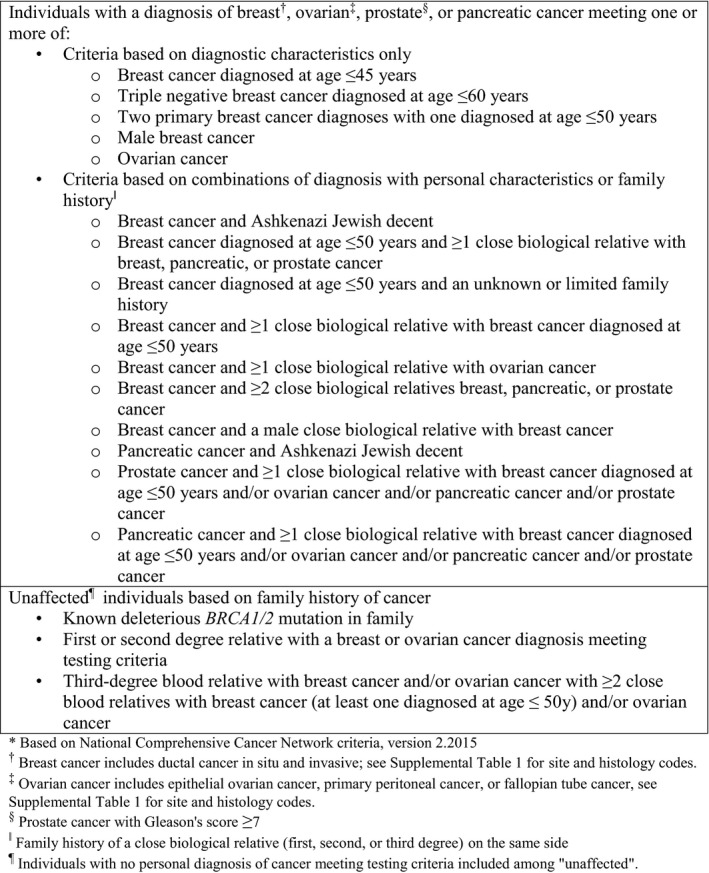
Hereditary breast and ovarian cancer syndrome testing criteria^*^

In order to estimate the prevalence of family history meeting HBOC testing criteria, we used data from the Utah Cancer Registry, a state‐wide registry since 1966 and a participating registry in the SEER program since 1973, linked to the UPDB. UPDB is a unique research data resource that includes extensive multi‐generation genealogies linked to individual health data from state‐wide resources.^16^, ^17^, ^18^ We assessed the prevalence of meeting HBOC criteria at the time of diagnosis for individuals with a cancer diagnosis. Cases included in the analysis were those diagnosed in 2010‐2015 with invasive cancer of HBOC sites or DCIS of the breast. We further limited the analysis to individuals who were linked through UPDB to enough family members in the state to evaluate family cancer history. This was defined as at least three adult relatives living in Utah after 1966. We used registry variables as described above to evaluate whether the case met criteria for HBOC testing based on diagnostic characteristics at the time of diagnosis. We used UPDB information about cancer diagnoses among relatives, querying relevant diagnoses among first, second, and third degree relatives as shown in Figure 1, to evaluate whether the case met criteria for HBOC testing based on family cancer history. We evaluated whether Utah residents without a personal diagnosis of cancer (unaffected) meeting HBOC criteria met criteria for testing based on family history. We limited the analysis to individuals who were living in Utah in 2018 and who were linked through UPDB to at least three adult relatives living in Utah after 1966. We again used UPDB information about cancer diagnoses among first, second, and third degree relatives to assess eligibility for HBOC testing as shown in Figure 1 under "unaffecteds." (We had no data source to evaluate the criterion "known deleterious BRCA 1/2 pathogenic variant in family".)

The study was approved by both the Institutional Review Boards of the University of Utah and Intermountain Healthcare.

### 2.2 Statistical analysis

We conducted descriptive analysis tabulating incident breast and ovarian cancer cases by the presence or absence of cancer characteristics meeting specific NCCN criteria and according to characteristics of the case at diagnosis including race, ethnicity, and place of residence. SEER*Stat software was used. For analyses incorporating family history criteria, a data set consisting of Utah Cancer Registry data linked by UPDB to family history variables was created and analyzed using SAS v9.2.

## 3 RESULTS

### 3.1 Breast and ovarian cancer cases meeting testing criteria based on diagnostic characteristics

From 2010 to 2015, 426,972 breast cancers (including invasive cancers and ductal carcinoma in situ) were diagnosed in the 18 SEER regions (Table 1). Based on diagnostic characteristics in the SEER data, overall 18.1% (n = 77 487) of breast cancer cases met one or more NCCN criteria for genetic testing. Among the female cases (n = 423 795), 12.2% (n = 51 631) met criteria due to being diagnosed at or below the age of 45. Young age at diagnosis was most prevalent among Hispanics (20.4%), followed by Asian or Pacific Islander (17.7%), with non‐Hispanic whites having the lowest proportion diagnosed under 45. The criterion of triple negative (ER/PR/Her2) breast cancer diagnosed in a woman younger than 60 years of age was met by 4.9% of female cases overall, and at a higher proportion (10.0%) in black or African American cases. Only 2.8% of cases met testing criteria based on a second breast cancer diagnosis with one at or before age 50. A diagnosis of male breast cancer occurred in 3,177 individuals, accounting for 0.7% of breast cancer cases.

**Table 1:** Population‐based prevalence of cancer cases with diagnostic characteristics meeting criteria for hereditary breast and ovarian cancer (HBOC) testing at the time of diagnosis, 18 SEER registries 2010‐2015

	Total		Race and ethnicity^a^											
			Non‐Hispanic White		Hispanic		Black or African American		Asian or Pacific Islander		American Indian or Alaska native		Other or unknown	
	n	%	n	%	n	%	n	%	n	%	n	%	n	%
Breast cancer, total	426 972		291 144		46 320		47 442		36 813		2303		2950	
Meets any criterion for testing^b^														
Yes	77 487	18.1	43 865	15.1	12 404	26.8	11 766	24.8	8473	23.0	459	19.9	520	17.6
No	349 485	81.9	247 279	84.9	33 916	73.2	35 676	75.2	28 340	77.0	1844	80.1	2430	82.4
Sex														
Male	3177	0.7	2307	0.8	230	0.5	461	1.0	146	0.4	7	0.3	26	0.9
Female	423 795	99.3	288 837	99.2	46 090	99.5	46 981	99.0	36 667	99.6	2296	99.7	2 924	99.1
Among female breast cancer cases														
Age at dx														
≤45	51 631	12.2	28 019	9.7	9405	20.4	6962	14.8	6492	17.7	335	14.6	418	14.3
46‐50	43 205	10.2	26 116	9.0	6360	13.8	5 267	11.2	4866	13.3	265	11.5	331	11.3
51+	328 959	77.6	234 702	81.3	30 325	65.8	34 752	74.0	25 309	69.0	1696	73.9	2175	74.4
Triple negative														
Yes	38 235	9.0	23 043	8.0	4587	10.0	7634	16.2	2601	7.1	215	9.4	155	5.3
Yes and dx ≤ age 60	20 926	4.9	11 243	3.9	3231	7.0	4683	10.0	1547	4.2	132	5.7	90	3.1
Yes and dx >age 60	17 309	4.1	11 800	4.1	1 356	2.9	2 951	6.3	1054	2.9	83	3.6	65	2.2
No	355 402	83.9	246 098	85.2	37 886	82.2	35 842	76.3	31 536	86.0	1932	84.1	2108	72.1
Missing	30 158	7.1	19 696	6.8	3617	7.8	3505	7.5	2530	6.9	149	6.5	661	22.6
Second breast primary														
Yes	40 412	9.5	28 690	9.9	3504	7.6	4 577	9.7	3350	9.1	207	9.0	84	2.9
Yes First primary ≤ age 50	11 761	2.8	7366	2.6	1423	3.1	1 639	3.5	1251	3.4	60	2.6	22	0.8
Yes First primary >age 50	28 651	6.8	21 324	7.4	2081	4.5	2938	6.3	2099	5.7	147	6.4	62	2.1
No	383 383	90.5	260 147	90.1	42 586	92.4	42 404	90.3	33 317	90.9	2089	91.0	2 840	97.1
Ovarian, fallopian tube, or primary peritoneal cancer, total	43 476		30 062		5638		3744		3579		275		178	
Meets any criterion for testing^c^														
Yes	35 479	81.6	25 141	83.6	4334	76.9	2788	74.5	2870	80.2	223	81.1	123	69.1
No	7997	18.4	4921	16.4	1304	23.1	956	25.5	709	19.8	52	18.9	55	30.9
Ovarian cancer histology														
Epithelial, mucinous	373	0.9	234	0.8	60	1.1	24	0.6	50	1.4	<5	0.4	<5	2.2
Epithelial, non‐mucinous	29 045	66.8	20 405	67.9	3620	64.2	2338	62.4	2403	67.1	182	66.2	97	54.5
Non‐epithelial	7 997	18.4	4921	16.4	1304	23.1	956	25.5	709	19.8	52	18.9	55	30.9
Fallopian tube or primary peritoneal	6 061	13.9	4502	15.0	654	11.6	426	11.4	417	11.7	40	14.5	22	12.4

a Includes Hispanic or Latino, of any race.

b "Yes" if male, diagnosed ≤age 45, triple negative and diagnosed ≤age 60, or second breast primary. Rows may not sum to total because a case may meet more than one criterion.

c "Yes" if epithelial ovarian or any fallopian tube, or primary peritoneal cancer.

During the same 2010‐2015 timeframe, 43 476 women were diagnosed with ovarian, fallopian tube, or primary peritoneal cancer. Of these, only non‐epithelial ovarian cancers do not meet criteria for genetic testing, and the remaining 35 479 (81.6%) do meet NCCN criteria. Non‐Hispanic White had the highest rate of ovarian cancers that met testing criteria (83.6%), followed by American Indian or Alaska Native (81.1%) and Asian or Pacific Islander (80.2%).

### 3.2 Utah cancer cases meeting diagnostic criteria for genetic testing based on family history

**Figure 2:**
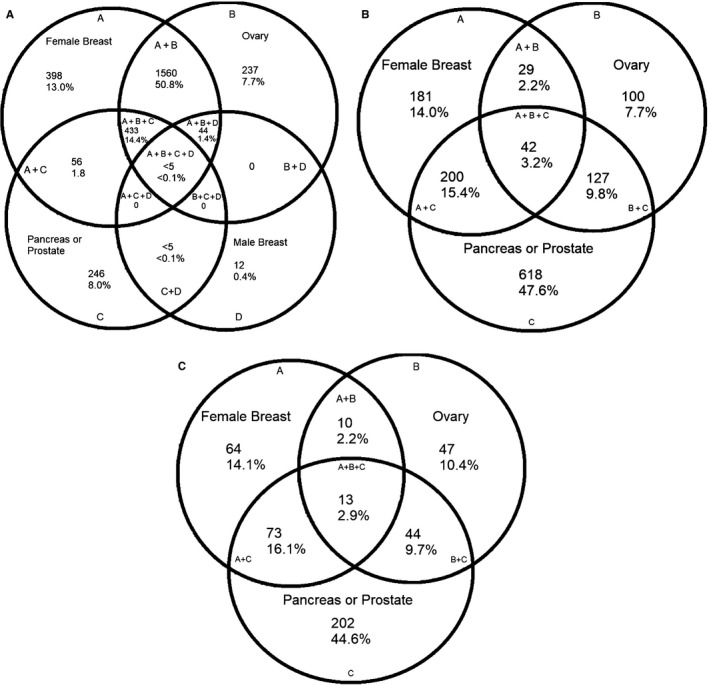
Cancer sites among relatives who contribute to family cancer history for incident cancer cases with a diagnosis of breast (A), prostate (B), or pancreatic (C) cancer, Utah 2010‐2015

There were 5590 total breast cancer cases diagnosed in Utah from 2010 to 2015 with evaluable family history. Breast cancer cases who met criteria for family history and/or diagnostic criteria comprised the majority, 62.9% of breast cancers in the population (Table 2). Of the total breast cancer cases, 21.6% (n = 1207) met diagnostic criteria for genetic testing. When UPDB pedigree information was included, 54.9% (n = 3068) of breast cancer cases met NCCN testing family history criteria. Breast cancer cases with a family history of solely breast cancer comprised 13% (n = 398) of the cases meeting family history criteria (Figure 2A). Others met criteria based on ovarian cancer family history only or combinations of breast or ovarian with male breast, prostate (Gleason ≥7), and pancreatic cancers.

**Table 2:** Prevalence of cancer cases with diagnostic characteristics or family history meeting hereditary breast and ovarian cancer (HBOC) testing criteria at the time of diagnosis, Utah^a^ 2010‐2015

	Total		Race and Ethnicity^b^					
			Non‐Hispanic White		Hispanic		Other	
	n	%	n	%	n	%	n	%
Breast cancer, total	5590		5353		144		93	
Meets any criterion for testing^c^								
Yes	3515	62.9	3409	63.7	70	48.6	36	38.7
No	2075	37.1	1944	36.3	74	51.4	57	61.3
Meets criteria based on diagnostic characteristics^d^								
Yes	1207	21.6	1137	21.2	49	34.0	21	22.6
No	4383	78.4	4216	78.8	95	66.0	72	77.4
Meets criteria based on family history								
Yes	3068	54.9	3006	56.2	39	27.1	23	24.7
No	2522	45.1	2347	43.8	105	72.9	70	75.3
Ovarian, primary peritoneal, or fallopian tube cancer, total	539		516		19		<5	
Meets any criterion for testing^e^								
Yes	506	93.9	485	94.0	17	89.5	<5	100.0
No	33	6.1	31	6.0	<5	10.5	0	0.0
Prostate cancer, total	5335		4997		106		232	
Meets criteria based on Gleason ≥7 and family history								
Yes	1297	24.3	1248	25.0	9	8.5	40	17.2
No, Gleason ≥7 but no family history	1590	29.8	1492	29.9	42	39.6	56	24.1
No, Gleason <7	2448	45.9	2257	45.2	55	51.9	136	58.6
Pancreatic cancer, total	888		824		45		19	
Meets criteria based on family history								
Yes	453	51.0	439	53.3	10	22.2	<5	21.1
No	435	49.0	385	46.7	35	77.8	15	78.9

a Table limited to cases who had ≥3 adult relatives living in Utah for evaluation of family history.

b Hispanic includes Hispanic or Latino, of any race. Other includes Black or African American, Asian or Pacific Islander, American Indian or Alaska Native, and other or unknown.

c "Yes" if meets diagnostic criteria or family history criteria. Rows may not add to total because a case may meet more than one criterion.

d "Yes" if male, diagnosed ≤age 45, triple negative and diagnosed ≤age 60, or second breast primary.

e "Yes" if epithelial ovarian or any fallopian tube or primary peritoneal cancer.

Family history criteria may also indicate that genetic testing for HBOC is appropriate for individuals with a diagnosis of prostate or pancreatic cancer. The Utah data indicate that 24.3% of prostate cancers and 51.0% of pancreatic cancers met any NCCN testing criteria at the time of diagnosis (Table 2). Although NCCN guidelines now have broader diagnostic criteria for prostate and pancreatic cancer referral, family history of pancreas or prostate cancer was the largest contributor to meeting criteria for both of these cancer sites because diagnostic criteria for these cancers were not included in the version of the guidelines used for this study. (Figure 2C,D).

### 3.3 Unaffected individuals in Utah who meet criteria for genetic evaluation

Individuals with no personal cancer diagnosis meeting HBOC criteria can be recommended for testing if they are a relative of an individual(s) with certain cancer diagnoses. In UPDB, about 1.7 million living individuals (from a state population of approximately 3 million) were evaluable for family history. In total, 11.6% (n = 197 601) of the unaffected, evaluated Utah population met HBOC criteria for genetic testing based on family history (Table 3). The most frequent contributing factor was a second‐degree relative with a cancer diagnosis meeting a criterion.

**Table 3:** Prevalence of unaffected individuals with family history meeting hereditary breast and ovarian cancer (HBOC) testing criteria, Utah

	n	%
Total population evaluated^a^	1 702 300	
Total unaffecteds meeting testing criteria based on family history^b^	197 601	
First degree relative with breast or ovarian cancer meeting testing criteria^c^	66 660	33.7
Second degree relative with breast or ovarian cancer meeting testing criteria^c^	111 161	56.3
≥3 relatives with breast or ovarian cancer^d^	41 051	20.8

a Adults living in Utah with no personal diagnosis of cancer meeting testing criteria and who have>=3 adult relatives in Utah to assess family cancer history.

b Total who meet testing criteria based on one or more of the family history criteria below. Rows may not add to total because a case can meet more than one criterion.

c Relative has a cancer diagnosis meeting testing criteria: breast cancer diagnosed ≤age 45, triple negative and diagnosed ≤age 60, second breast primary if first was diagnosed ≤age 50, or male; epithelial ovarian cancer or any primary peritoneal or fallopian tube cancer.

d At least 3 close blood relatives with breast and/or ovarian cancer including one relative with breast cancer diagnosed ≤age 50.

## 4 DISCUSSION

Given the limited availability of objectively determined family cancer history data,^12^ predicting the prevalence of individuals in the general population who will meet genetic testing criteria is a significant challenge. In this study, utilizing a database that incorporates cancer registry data with extensive genealogies allows the first estimate of a state's prevalence of individuals in the general population who meet HBOC criteria. This was measured using both a snapshot approach for individuals with a diagnosis of cancer in 2010‐2015, and by identifying living individuals who meet HBOC criteria. We found that more than 50% of individuals diagnosed with breast, ovarian, or pancreatic cancer meet criteria at the time of diagnosis. This is higher than previous studies that have shown approximately 35.6% of breast cancer patients meeting NCCN criteria;^19^ however, these were based on in‐home family interviews, not objective cancer data. We estimate that 11.6% of the unaffected Utah population meets criteria for genetic testing for *BRCA1* and *BRCA2* pathogenic variants based on family history extended out to second degree relatives.

Although NCCN guidelines present testing criteria for unaffected individuals, they recommend that genetic testing begin with an affected case as feasible.^20^ This both minimizes potentially unnecessary testing expense and provides the most accurate risk assessment for a family. Given that family members (with the exception of monozygotic twins) are at most 50% genetically similar, testing an affected patient has the highest likelihood of identifying a genetic susceptibility for their cancer diagnosis. Negative genetic testing in an unaffected first‐degree relative can be uninformative, as it will remain unknown whether the affected patient had a genetic susceptibility to their cancer diagnosis. Furthermore, unaffected relatives with negative germline testing often undergo increased cancer screening that may unnecessary if they were found to be “true negatives” when an affected relative has a pathogenic variant identified. If an affected individual tests negative, genetic testing is not indicated for their relatives, which allows for optimal usage of health care resources. A limitation of the present study is that genetic testing status and results were not available. Therefore, it is presumed a portion of those found to meet criteria for testing in this study do not need genetic testing given previous family member testing and/or previous testing for themselves.

Quantifying the need for genetic testing can also have implications for clinician staffing and conversations with patients during appointments. Identifying cases that need genetic evaluation lies in the oncologists’ realm, however, other clinicians can oversee the risk assessment and genetic testing portion. Genetic counselors can be utilized to facilitate the genetic testing process for this large population, notably in cancer institutes who see high volumes of HBOC‐related cancers that would meet criteria for genetic testing.

Since our initial analysis, NCCN guidelines have expanded to recommend genetic testing for all patients with pancreatic cancer or metastatic prostate cancer regardless of family history.^20^, ^21^ Given these ever‐expanding guidelines, it is expected that analysis performed using updated guidelines would show pancreatic cancer having similar case proportions to the ovarian cancer analyses performed. Similarly, the proportion of prostate cancer cases meeting criteria will be significantly increased. There is also evidence that patients who do not meet current referral criteria may also have pathogenic variants and would benefit from testing. This results in an expected increase in individuals meeting criteria for genetic services, which must be addressed on multiple levels.

In general, identifying the most appropriate unaffected patients to maximize genetic testing impact is typically a responsibility of primary care providers and other physicians. Given that over 10% of primary care providers' patient cohort is indicated for genetic testing, and up to 60% of an oncologists' patient cohort, family health history remains an important part of the initial intake. Asking patients about cancers in the family across all specialties, along with type and age of diagnosis, can allow for a comprehensive health history to facilitate genetic testing in appropriate patients.

Ultimately, better identification of individuals who meet genetic testing criteria, paired with expanded guidelines, will dramatically drive demand for genetic services. Considering options for genetic counseling and information service delivery will be crucial to meet this demand. From training a growing genetic counseling workforce, to partnering with non‐genetics providers to provide collaborative genetics education, providers across various specialties, not limited to primary care and genetic counselors, must come together to increase appropriate identification and provision of genetic services to individuals. Policy‐level considerations that address geographic and socioeconomic disparities will also be imperative to ensure widespread access to genetic services.

This study is a snapshot of one state and may be an over or underestimate of the true national population that meets HBOC criteria. Utah has the highest average number of children per household in the country (2.21, national median 1.85), and with larger family sizes, the proportion of unaffected individuals who have a relative with a cancer diagnosis may be somewhat larger than in other US states and therefore the Utah estimates may overestimate the number of at‐risk relatives compared to other geographic areas. UPDB captures cancer diagnoses only for Utah residents; to the extent that Utah residents have relatives who reside in other states, the family history may be underestimated. In addition, prevalence may be higher in states with larger populations of Ashkenazi Jewish descent given their increased incidence of *BRCA1/2* pathogenic variants. Limitations aside, this is the first study to utilize objective family cancer history and diagnostic criteria from a SEER cancer registry to quantify the percentage of individuals in a state meeting NCCN HBOC criteria.

Overall, this study highlights the large proportion of a state's population that may qualify for genetic testing, although it is limited in that it does not account for those who have undergone genetic evaluation. Efforts focused on identifying affected cases can have the greatest impact on resource utilization and efficiently evaluating this population. Given the high proportion of breast, pancreatic, prostate, and ovarian cases that meet criteria due to family history, there is a need for heightened awareness of family health history. In families where testing the affected case is not possible, evaluation of the unaffected population to identify high‐risk individuals who may benefit from HBOC genetic testing is necessary.

## ACKNOWLEDGMENTS

The study was supported by the Enhancing Cancer Genomic Best Practices through Education, Surveillance and Policy Grant, DP005360‐01, funded by the Center for Disease Control and Prevention; contents are solely the responsibility of the authors and may not represent the official views of the Centers for Disease Control and Prevention or the Department of Health and Human Services. The Utah Cancer Registry is funded by the National Cancer Institute's SEER Program, Contract No. HHSN261201800016I, the US Center for Disease Control and Prevention's National Program of Cancer Registries, Cooperative Agreement No. NU58DP0063200‐01, with additional support from the University of Utah and Huntsman Cancer Foundation. The UPDB and Genetic Counseling Shared Resource are supported in part by grant P30 CA2014 by the National Cancer Institute and awarded to the Huntsman Cancer Institute and the Huntsman Cancer Foundation. The study sponsor was involved in the manuscript preparation as it relates to contributing to the background and facilitating meetings amongst the team. The UPDB is also supported by the University of Utah's Program in Personalized Health and Center for Clinical and Translational Science. The authors thank all stakeholders across multiple Utah institutions who have played a key role in this grant's mission and activities.

## CONFLICT OF INTEREST

Samantha Greenberg reports one‐time consulting fees from Tempus laboratories, outside of the scope of submitted work. The rest of the authorship reports no conflict of interests.

## AUTHOR CONTRIBUTIONS

Samantha Greenberg contributed to data curation, formal analysis, writing‐original draft, and writing‐review, and editing. Saundra S. Buys contributed to conceptualization, methodology, project administration, supervision, validation, writing‐review, and editing. Sandie L. Edwards contributed to conceptualization, data curation, formal analysis, investigation, methodology, project administration, resources, validation, writing‐original draft, writing‐review and editing. Whitney Espinel contributed to supervision, writing‐original draft, writing‐review and editing. Alison Fraser contributed to conceptualization, data curation, formal analysis, software. Amanda Gammon contributed to conceptualization, data curation, writing‐review and editing. Brent Hafen contributed to conceptualization, methodology, supervision. Kim A. Herget contributed to data curation, formal analysis, methodology, validation, writing‐review and editing. Wendy Kohlmann contributed to conceptualization, data curation, supervision, writing‐review and editing. Camille Roundy contributed to conceptualization, project administration, resources, supervision, writing‐original draft, writing‐review and editing. Carol Sweeney contributed to conceptualization, data curation, formal analysis, investigation, methodology, project administration, resources, supervision, writing‐original draft, writing‐review and editing.

## Data Availability

Data sharing is not applicable to this article as no new data were created or analyzed in this study.
