# Supplementary material for: Population prevalence of individuals meeting criteria for hereditary breast and ovarian cancer testing
Source: Cancer Med. 2019 Sep 18;8(15):6789–98. doi: 10.1002/cam4.2534 (PMC6825998; doi:10.1002/cam4.2534)
Supplement: Supplementary file 1 [file CAM4-8-6789-s001.docx]

Supplemental table 1. Eligible cancer diagnoses, cancer site code, histology, and other criteria

|  | Site codes (ICD-03) | Other inclusion criteria | Histology type exclusions (ICD-03) |
| --- | --- | --- | --- |
| Cancer diagnoses for affected (i.e. cancer proband) |  |  |  |
| Breast, in situ | C50.0–C50.9 | In situ, ductal histologies 8500/2, 8523/2, 8230/2, 8522/2, 8507/2, 8503/2, 8543/2, 8540/2, 8453/2, 8521/2, 8541/2 | 9050-9055, 9140, or 9590-9992 |
| Breast, invasive | C50.0–C50.9 |  | 9050-9055, 9140, or 9590-9992 |
| Ovarian, invasive epithelial | C56.9 | Epithelial, nonmucinous histologies 8001, 8010, 8020, 8021, 8041, 8050, 8070, 8071, 8075, 8120, 8140, 8246, 8260, 8310, 8323, 8380, 8381, 8440, 8441, 8442, 8460, 8461, 8462, 8490, 8950, 8980, 9000  Epithelial mucinous histologies 8470, 8472, 8480 | Non epithelial histologies 8000, 8002, 8012, 8013, 8032, 8033, 8046, 8052, 8074, 8130, 8144, 8230, 8240, 8243, 8255, 8313, 8337, 8340, 8344, 8382, 8383, 8401, 8410, 8450, 8463, 8471, 8481, 8482, 8504, 8507, 8560, 8562, 8570, 8590, 8593, 8600, 8620, 8622, 8631, 8634, 8640, 8650, 8670, 8800, 8804, 8806, 8810, 8890, 8900, 8930, 8931, 8933, 8935, 8940, 8951, 8981, 9014, 9060, 9064, 9065, 9071, 9080, 9084, 9085, 9090, 9091, 9100, 9110, 9120, 9220, 9260, 9391, 9473  SEER standard exclusions: 9050-9055, 9140, or 9590-9992 |
| Primary Peritoneal | C48.1-C48.2 |  | 9050-9055, 9140, or 9590-9992 |
| Fallopian Tube | C57.0 |  | 9050-9055, 9140, or 9590-9992 |
| Prostate | C61.9 | Gleason’s score >=7 defined as grade ≥3 for any diagnosis date, CS Site Specific Factor 6 ≥007 for diagnosis years 2004-2009, or CS Site Specific Factor 8 ≥007 for diagnosis years 2010-2015 | 9050-9055, 9140, or 9590-9992 |
| Pancreas | C25.0-C25.9 |  | 9050-9055, 9140, or 9590-9992 |
| Cancer diagnoses for relatives |  |  |  |
| Breast, in situ | C50.0–C50.9 | In situ, ductal histologies 8500/2, 8523/2, 8230/2, 8522/2, 8507/2, 8503/2, 8543/2, 8540/2, 8453/2, 8521/2, 8541/2 | 9050-9055, 9140, or 9590-9992 |
| Breast, invasive | C50.0–C50.9 |  | 9050-9055, 9140, or 9590-9992 |
| Ovarian, invasive epithelial | C56.9 | Epithelial, nonmucinous histologies 8001, 8010, 8020, 8021, 8041, 8050, 8070, 8071, 8075, 8120, 8140, 8246, 8260, 8310, 8323, 8380, 8381, 8440, 8441, 8442, 8460, 8461, 8462, 8490, 8950, 8980, 9000  Epithelial mucinous histologies 8470, 8472, 8480 | Non epithelial histologies 8000, 8002, 8012, 8013, 8032, 8033, 8046, 8052, 8074, 8130, 8144, 8230, 8240, 8243, 8255, 8313, 8337, 8340, 8344, 8382, 8383, 8401, 8410, 8450, 8463, 8471, 8481, 8482, 8504, 8507, 8560, 8562, 8570, 8590, 8593, 8600, 8620, 8622, 8631, 8634, 8640, 8650, 8670, 8800, 8804, 8806, 8810, 8890, 8900, 8930, 8931, 8933, 8935, 8940, 8951, 8981, 9014, 9060, 9064, 9065, 9071, 9080, 9084, 9085, 9090, 9091, 9100, 9110, 9120, 9220, 9260, 9391, 9473  SEER standard exclusions: 9050-9055, 9140, or 9590-9992 |
| Primary Peritoneal | C48.1-C48.2 |  | 9050-9055, 9140, or 9590-9992 |
| Fallopian Tube | C57.0 |  | 9050-9055, 9140, or 9590-9992 |
| Prostate | C61.9 | Gleason’s score >=7 defined as grade ≥3 for any diagnosis date, CS Site Specific Factor 6 ≥007 for diagnosis years 2004-2009, or CS Site Specific Factor 8 ≥007 for diagnosis years 2010-2015 | 9050-9055, 9140, or 9590-9992 |
| Pancreas | C25.0-C25.9 |  | 9050-9055, 9140, or 9590-9992 |
